# Assessment of Apple Peel Barrier Effect to Pesticide Permeation Using Franz Diffusion Cell and QuEChERS Method Coupled with GC-MS/MS

**DOI:** 10.3390/foods12173220

**Published:** 2023-08-27

**Authors:** Maciej Tankiewicz

**Affiliations:** Department of Environmental Toxicology, Faculty of Health Sciences with Institute of Maritime and Tropical Medicine, Medical University of Gdańsk, Dębowa Str. 23A, 80-204 Gdańsk, Poland; maciej.tankiewicz@gumed.edu.pl; Tel.: +48-58-349-19-34; Fax: +48-58-349-19-37

**Keywords:** pesticides, permeation, apple peel, Franz diffusion cell, QuEChERS, GC-MS/MS

## Abstract

In this study, a new approach to pesticide permeation through the apple peel into the pulp is discussed. The tested compounds can be classified, based on mode of action, as systemic (boscalid, cyprodinil, pirimicarb, propiconazole and tebuconazole) or contact (captan, cypermethrin and fludioxonil) pesticides. The barrier effect was assessed using a Franz flow-type vertical diffusion cell system. A residue analysis was performed using a modified quick, easy, cheap, efficient, rugged and safe (QuEChERS) extraction method coupled to gas chromatography with tandem mass spectrometry (GC-MS/MS). The limits of detection (LODs) ranged between 2.6 µg kg^−1^ (pirimicarb) and 17 µg kg^−1^ (captan), with the coefficient of variability (CV) lower than 6%, while recoveries ranged from 85% (boscalid) to 112% (captan) at 0.1 and 1 mg kg^−1^ spiked levels. The highest peel penetration was observed for pirimicarb, captan and cyprodinil, with cumulative permeations of 90, 19 and 17 µg cm^−2^, respectively. The total absorption was in the range from 0.32% (tebuconazole) to 32% (pirimicarb). Only cypermethrin was not quantitatively detected in the pulp, and its use can be recommended in crop protection techniques. The obtained results indicate that molecular weight, octanol-water partition coefficient and water solubility are important parameters determining the process of pesticide absorption.

## 1. Introduction

Modern technologies of food production and management are aimed at improving its quality and safety and extending its shelf life. This applies especially to fresh fruits and vegetables as they are a good source of vitamins, minerals and fibers, but their cultivation and storage require the use of pesticides [1,2]. During the process of plant growth, they are used to increase crop yields and to prevent plant diseases and pest infestations [3]. This leads to the presence of pesticides in agricultural products that may pose a potential threat to consumer health [1,4]. According to the latest European Union (EU) report on pesticide residues in food, 40.3% of the analyzed samples contained quantified residues (35,485 samples out of 88,141). In total, maximum residue levels (MRLs) were exceeded in 5.1% of the samples (4475 samples), an increase compared with 2019 (3.9%) [5]. This confirms the need for studies to ensure the quality and safety of food available on the market.

Consumers’ awareness in this regard is increasing, and various methods of reducing exposure to pesticides are commonly used. One of the most popular methods is peeling. This applies primarily to apples, which are most often served to children in this form [6]. Other food processing methods include washing, boiling, blanching, juicing, cold storage, etc. [7,8,9]. In addition, more advanced techniques such as cold plasma, irradiation and pulsed electric fields have been proposed in the literature [10,11]. Some of the traditional methods have shown a high level of removal efficiency but mainly for pesticides adsorbed on the peel or absorbed in the peel [12,13]. For example, the effectiveness of washing and peeling has been tested on some fruits and vegetables that are consumed daily, such as tomatoes, cucumbers, apples and grapes [14]. While these techniques were effective in removal, the treated products lost some or most of their nutritional value at the same time. Therefore, other solutions are needed to help reduce exposure and ensure adequate food safety. It should be noted that pesticides that penetrate through the peel into the pulp could accumulate and contaminate the food, and the commonly used removal methods may not be sufficient as they mainly deal with residues in the peel but not in the pulp. 

Due to the diverse composition of fruits and vegetables, various sizes (molecular weight) and physicochemical properties of the pesticides used, there may be different mechanisms of sorption and permeation into the pulp [15]. It is thought that wax and cutin are the main participants in the sorption of non-polar organic contaminants into the peel, and the hydrophobic effect is the dominant permeation mechanism. Polysaccharides (cellulose and pectin) and polar ingredients, on the other hand, play a negative role in their permeation, favoring the sorption of polar compounds instead [16,17]. Some studies have proposed different hypotheses of sorption reaction kinetics, mainly depending on the nature and presence of specific chemical substances in the peel [18,19,20]. In addition, other studies can be found in the literature on the exposure of fruit peels to pesticides and their ability to permeate into the pulp [20,21,22]. Most often, the products were immersed or washed with pesticide solutions, and then after peeling, residue levels in the peel and subsequent layers of pulp were determined, assessing the permeability of the relative pesticide. However, crop exposure to pesticides is mainly through spraying, therefore, permeation results can vary due to different contact conditions.

Pesticide permeation studies are crucial to understanding sorption processes and protecting fruits and vegetables from contamination [23], and they can be used to create a recommendation list of compounds that do not accumulate in the pulp and act as intended. This can help to minimize consumer exposure to residues, and they can be safely and effectively removed from food using known techniques. Additionally, they allow the assessment of whether the residue in the pulp after, e.g., peeling may pose a threat to consumers. This is particularly important as whole products, including the peels, are currently used for residue determination [24]. The degree of pesticide penetration may be one of the factors affecting the persistence of the pesticide over time and the amount of its residues [25]. 

The aim of this study was to assess the permeation of a series of pesticides, currently approved for agricultural use (six fungicides and two insecticides), through an apple peel. These are of great importance in increasing the efficiency and quality of food production. The compounds in question are regularly detected in apples during monitoring studies conducted by public monitoring services [5], as shown in our previous research [26,27]. Moreover, these pesticides are most often applied via spraying and thus come into direct contact with the peel of apples treated this way. In this study, an attempt was made to check whether the compounds most often present in the pulp could penetrate through the peel. However, it should be emphasized, that the peel is only one of several pathways of pesticide permeation into the pulp. For peel testing, a Franz flow-type vertical diffusion cell system was used. This method is commonly used for the transdermal testing of topical drugs and liquid or semi-liquid dosage forms of pharmaceuticals and cosmetics [28]. Their use is recommended in pharmacopoeial methods and guidance documents of the Organization for Economic Co-operation and Development (OECD) and the Scientific Committee on Consumer Safety (SCCS) of the EU [29,30]. According to the guidelines of the United States Food and Drug Administration (U.S. FDA), they are appropriate for transdermal examinations because they ensure a vertical flow of the medium (acceptor fluid), allowing for constant monitoring of parameters corresponding to real-life conditions [31]. In addition, the temperature control and precise measurement of defined volumes of the medium is fairly simple and straightforward, leading to reproducible test results [32]. To the best of our knowledge, this is the first study applying this method to assess the barrier effect of fruit peels. The use of a plant fragment as a (semi-permeable) membrane and the composition of the acceptor medium, which reflects the composition of the pulp and the morphological structure of the tested crop, are innovative. The results of this study may be useful in protecting public health by minimizing the health risks of consumers and their exposure to pesticide residues.

## 2. Materials and Methods

### 2.1. Reagents and Materials

Active ingredients of pesticides with purity >95.5% (boscalid, captan, cypermethrin, cyprodinil, fludioxonil, pirimicarb, propiconazole and tebuconazole) were purchased from AccuStandard Inc. (New Haven, CT, USA) and used to prepare stock standard solutions (5 mg mL^−1^) in acetonitrile (ACN). For diffusion studies, apple peels were sprayed with 100 µL of each standard solution. ACN and methanol (99.9%) for chromatography were obtained from Merck KgaA (Darmstadt, Germany). Ultrapure water was prepared using a Hydrolab water purification system (Hydrolab, Straszyn, Poland). All solvents and chemicals used in this study were of analytical grade. 

Isolation of permeated pesticides and extract purification were performed using the quick, easy, cheap, efficient, rugged and safe (QuEChERS) method. Currently, this is one of the most popular extraction techniques for the preparation of fruit and vegetable samples, recommended in the official methods [2,4,8,12,33]. For this purpose, the pre-weighted extraction kits (containing 400 mg of anhydrous magnesium sulfate (MgSO_4_), 100 mg of sodium chloride (NaCl), 50 mg of disodium citrate (Na_2_Cit), 100 mg of trisodium citrate (Na_3_Cit)) and purification kits based on dispersive solid phase extraction (dSPE) (containing of 150 mg of MgSO_4_ and 25 mg of primary secondary amine (PSA)) were purchased from United Chemical Technologies, Inc. (Bristol, PA, USA). 

Standard mixtures at 100 mg L^−1^ and 1 mg L^−1^ were prepared in ACN via dilution of the corresponding stock standard solutions and stored at −20 °C in a freezer. They were used to spike apple samples for calibration purposes and recovery studies in the concentration range from 0.01 to 10 mg kg^−1^. The calibration standards at concentrations of 0.01, 0.05, 0.1, 1.0 and 10 mg kg^−1^ were prepared via addition of the standard mixtures directly to the matrix. 

A 40× magnification Nikon Eclipse TS100 F inverted microscope with a DeltaPix Invenio 5 S1 camera (Nikon Instruments Europe B.V., Amstelveen, The Netherlands) was used to image and control the continuity of apple peels. This ensured that the peels used as membranes in the permeation tests were not damaged after being cut from the apples. In addition, microscopic images were taken before and after each permeation test.

### 2.2. Pesticides’ Penetration Ability

The biological fate of pesticides in plant tissues is determined by their chemical properties and environmental conditions [3,8]. The chemical formula, substance group, type of action, human health issues, molecular weight and physicochemical properties of the studied pesticides are presented in Table S1 of the Supplementary Materials (SM) [34,35]. The molecular weights of the analytes range from 238.39 g mol^−1^ (pirimicarb) to 416.30 g mol^−1^ (cypermethrin). With regard to solubility in water, the tested pesticides fall within a wide range of solubility, going from very soluble compounds, such as pirimicarb (water solubility of 3100 mg L^−1^), to practically insoluble compounds like cypermethrin or fludioxonil (with solubility of 0.009 mg L^−1^ and 1.8 mg L^−1^, respectively). Similarly, the range of values for octanol–water partition coefficients (log P) is wide at pH 7 and 20 °C. For example, for cypermethrin it is 5.55, while for pirimicarb it is only 1.7. Such a large span of values proves that this group of compounds exhibits disparate properties and thus may have different penetration characteristics. Moreover, the pesticide mode of action may also have an effect on penetration ability [3,13]. Systemic pesticides (boscalid, cyprodinil, pirimicarb, propiconazole and tebuconazole) are usually hydrophilic, which is assessed using the acid dissociation constant (pKa) and log P. They can permeate into plant tissues and translocate through the peel of fruits and vegetables or plant epidermis. In vascular plants, they can shift across phloem or xylem transporting tissues. Non-systemic pesticides (captan, cypermethrin and fludioxonil) tend to be lipophilic and therefore migrate to the waxy layer covering the plant epidermis, making them resistant to washout by rain. They have little or no ability to permeate into plant tissues (leaves, roots or fruits). It should be emphasized that the above-mentioned penetration abilities relate to active substances and not to pesticide preparations containing other additives [25]. Their function is to increase the solubility of the preparation, sometimes allowing the active substance to be dissolved in water, improving the adhesion of the spray liquid to the leaves of the plant, improving the penetration of the active substance into the plant cells or preventing the spray liquid from foaming. Consequently, additives may allow the penetration of non-systemic compounds. Moreover, pesticide treatments in the field are usually not uniform and therefore the distribution of residues can vary significantly. This process can also be affected by the size, shape and density of the plants. The identification of the high variability of pesticide residues and the determination of the conditions of their maximum permeation into crops, leading to their contamination, is considered important in the assessment of acute dietary exposure.

### 2.3. Apple and Peel Samples Preparation

The apples (Szampion variety) used in the study were purchased each time at the same location at the local market in Gdansk (Poland) from domestic producers. They were collected daily in accordance with European Commission Directive 2002/63/EC and came from organic plantations [24]. Each batch of collected apples was divided into 3 parts: for the preparation of the acceptor medium, for the preparation of the peels to be used as membranes for testing, and for checking contamination with pesticide residues. Thus, positive falsification of the results was eliminated. Before testing, the apples were stored in the dark at 4 °C and transported to the lab.

From each batch of purchased fruit, a sample of 1 kg was weighed on a technical scale. They were then cut into quarters with a knife and ground into a homogeneous pulp using a Microtron MB550 laboratory homogenizer (Kinematica AG, Luzern, Switzerland). Subsequently, 400 mL of ultrapure water was added to the mixture and then filtered using paper filters. This step was necessary because apple particles can clog the Franz cell, precluding sampling during permeation testing [29]. The obtained apple filtrates were used as a representative matrix intended for validation of the research method and the acceptor medium in the permeation study. 

**Figure 1:**
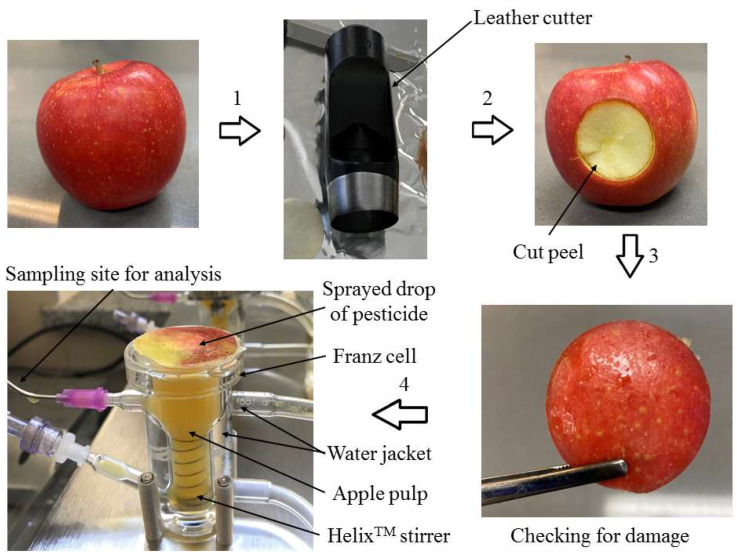
Scheme of procedure of apple peel preparation and performance of the permeability test for 24 h using a Franz flow-type vertical diffusion cell system (control samples were not sprayed with pesticides); 1—sample collection, 2—membrane preparation, 3—peel integrity testing and damage checking and 4—conducting a permeation test using the Franz cell.

The second part of each batch of apples was used to prepare the peels to be used as membranes. For this purpose, peels with a diameter of 30 mm were cut out with a circular leather cutter. The area of the examined peels was 7.1 cm^2^. Subsequently, most of the flesh from under the cuticles was gently removed up to 3–5 mm in thickness, so as not to damage the continuity of the peel barrier. The peels in question must not have been previously damaged, and the cut circle shape must not have been deformed. Peel integrity and the presence of damage were evaluated under a magnifying glass. The method used for preparing the apple peel and performing the permeability test is presented in Figure 1.

### 2.4. Franz Cell Diffusion Tests

A 6-cell manual Franz vertical diffusion system coupled with a 6-place magnetic stirrer (2mag-AG, München, Germany) and heated circulating water bath (JULABO MA-4 GmbH, Seelbach, Germany) was obtained from Hanson Research (Chatsworth, CA, USA). All tests were carried out in 7 mL vertical diffusion cells with open cell top (1.5 cm diameter and 1.8 cm^2^ diffusion area), PTFE dosage wafer with O-ring (Strat-M^®^ membrane) and cap and clamp assembly [30,31,32]. An additional membrane (3 mm thickness) was used to seal the cells and prevent the peels from being crushed during the permeation tests. Temperature and agitation were set to 25 °C and 350 rpm, respectively.

For each series of diffusion experiments, fresh standard solutions, pulp filtrate and peels were prepared, and triple measurements were performed for every studied pesticide separately. Additionally, tests with blank samples without pesticides were carried out to check the matrix effect and eliminate false-positive results. Subsequently, the Franz cells were filled with apple pulp filtrate. The apple peel was then placed and covered with the cell top set. Finally, the cells were inspected to ensure that there were no air bubbles between the peel and the pulp. After preparation, a drop (100 µL) of the tested pesticide from the 5 mg mL^−1^ standard solution was applied on the top of the peel. Thus, 0.5 mg of the pesticide was dosed at one time. Its size was selected in terms of the sensitivity and linearity of the methodology, which allowed the determination of studied compounds at the MRL values. The maximum concentration in the case of 100% diffusion would be 71 mg kg^−1^. Aliquots (1 mL) were collected from the receptor sections at specified time intervals expressed in hours (0, 0.5, 1, 1.5, 2, 2.5, 3, 4, 6, 8, 16 and 24 h) by injecting pulp filtrate using a chromatographic syringe. Subsequently, samples were extracted and purified using the QuEChERS method and analyzed using gas chromatography coupled with tandem mass spectrometry (GC-MS/MS) [27]. For this purpose, the collected sample was transferred to a 4 mL conical vial, and 1 mL of ACN was added and vortex mixed for 1 min. Then, an aliquot of the extraction salt mixture was added, and it was vortex mixed again for 1 min and centrifuged for 10 min at 1800× *g*. Next, all upper layers were mixed with purification salts for dSPE and centrifuged for 10 min at 1800× *g*. A clear extract was obtained and transferred to 1.5 mL glass vials for further chromatographic analysis.

### 2.5. Chromatographic Analysis

Pesticide concentrations in apple pulp and in the third part of each batch of purchased apples were determined using gas chromatography (GC-2010 PLUS) equipped with an autosampler (AOC–20ia) and tandem mass spectrometer (MS-TQ8040) from Shimadzu Corp. (Kyoto, Japan). The separation was performed on GC Zebron^TM^ ZB-5 MSi column (30 m, 0.25 mm i.d. and 0.25 µm film thickness, Phenomenex, Torrance, CA, USA) with helium as the carrier gas (99.9999%, Air Products, Warsaw, Poland) and flow rate of 1 mL min^−1^. The oven temperature was programmed as follows: initially 70 °C, then 70–290 °C at 10 °C min^−1^ and held for 3 min. The injection temperature and volume were set to 250 °C and 3 µL, respectively. Samples were injected in the splitless mode at higher pressure conditions (150 kPa). The triple quadrupole mass spectrometer was operated in electron impact (EI) mode with 70 V of ionization voltage and 150 µA of emission current. The studied pesticides were determined in Q3 selected ion monitoring (SIM) mode. The transfer line, ion source and quadrupole temperatures were maintained at 310 °C, 220 °C and 150 °C, respectively. For the screening of pesticide residues in the analyzed apples, the extensive Smart Pesticides Database software (version 1.03, Shimadzu Corp., Kyoto, Japan) with MRM Optimization Tool was used.

### 2.6. Method Validation and Data Analysis

Only non-pesticide-containing apples were used in the validation studies to avoid adulteration. Apple samples and apple pulp filtrates were extracted according to Section 2.3 with the difference that the mass of the apple sample was 10 g, and 1 mL of pulp filtrate was taken for quantitative analysis of permeated pesticides. 

The proposed methodology was validated in accordance with the EU official guideline (SANTE) for pesticide residue control in food and feed [36]. In addition, the OECD guideline for the testing of chemicals (skin absorption: in vitro method) was adopted for this study [29]. The chosen parameters (linearity, limit of detection (LOD), limit of quantification (LOQ), precision, repeatability, matrix effect and recovery) were determined and used for method evaluation. Confirmation of pesticide identification was based on ion ratio statistics with a relative tolerance of up to 30%. The concentration level of the individual pesticides was calculated using 5-point matrix-matched calibration curve. Linearity was assessed on the basis of the coefficient of determination (R^2^). For calibration, extracts of blank matrices were also used. LODs and LOQs were calculated based on the standard deviations (SD) of detector responses for the lowest concentration level and the slope of the calibration curve (b) according to the formulas: LOD = 3.3 × (SD/b) and LOQ = 10 × (SD/b), respectively. Precision, expressed as a percentage of the relative standard deviation (RSD%), was determined by examining the retention times and peak areas of analytes for samples spiked at 0.1 and 1 mg kg^−1^ concentration levels, injected (with five replicates) twice per day and on two different days. A recovery study was carried out with five replicates at 0.1 and 1 mg kg^−1^ spiking levels. Repeatability was described with the coefficient of variability (CV %). 

The apple peel permeability (expressed in µg cm^−2^) was calculated from the quantity of pesticides in 7 mL of apple pulp, which permeated through the peel during the test duration, divided by the diffusion area. Next, the cumulative permeation factors after 24 h of spraying were calculated. Absorption flux was obtained from the cumulative permeation values divided by the duration of the test and expressed as µg cm^−2^ per hour. The total amounts of diffused compounds were compared with the EU regulation on pesticide MRLs in apples to assess the risk to consumers and the effectiveness of the peeling technique in removing residues [34,37]. Additionally, the total absorption rate for each tested pesticide was determined based on the ratio of the total amount in the apple pulp to the amount in the donor phase according to the formula: total absorption (%) = quantity of pesticide in the pulp/total amount in donor × 100. All calculations were performed using Microsoft Excel™ 2010 (USA). The statistical significance of the differences observed after triplicate assays was evaluated with the one-way analysis of variance (ANOVA) with the similarity Duncan’s test, using the STATISTICA, TIBCO Software Inc. (2020) Data Science Workbench, version 14. In all the calculations, the statistical significance level was set to *p* < 0.05.

## 3. Results and Discussion

### 3.1. Characteristics of the Apple Peel as a Membrane

**Figure 2:**
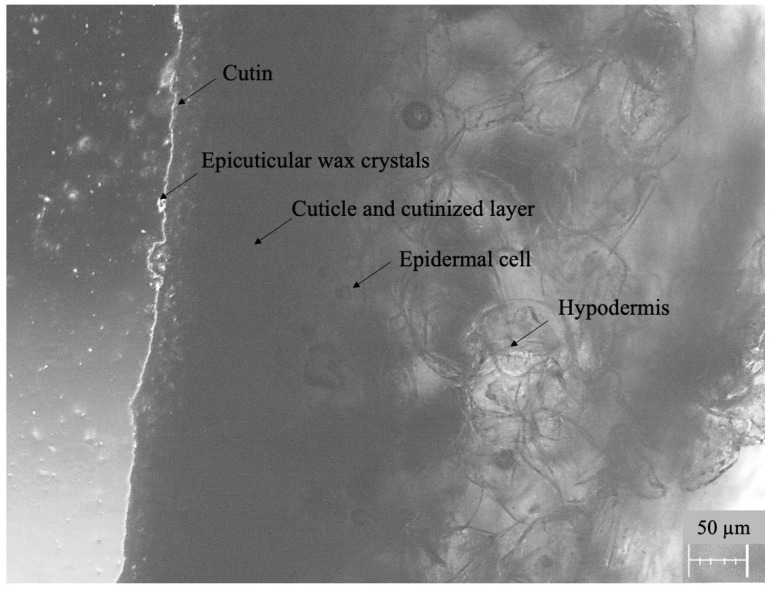
Microscopic image (magnification 40×) of a cross-section of an apple peel fragment with its composition before permeability test, obtained with a Nikon Eclipse TS100 F inverted microscope.

A microscopic image of a cross-section of an apple peel fragment with its composition is shown in Figure 2. As can be observed, the outer layer, commonly referred to as the peel, is composed of an *epidermis* covered by the cuticle and the multilayered *hypodermis* containing tangential or, less frequently, angular collenchyma cells [38]. The surface of the cuticle properly contains a layer of epicuticular waxes in the form of a continuous film of amorphous wax (cutin) and various forms of crystalline wax [39]. The cuticle, which is a lipid-type epithelium with a heterogeneous structure, has an important protective role as a barrier between the internal and external environment. The inner layer of the cuticle, adhering to the cell wall of the *epidermis*, forms the so-called cuticular layer, which is usually reticular and contains lipid substances and polysaccharides. The *epidermis* contributes to the plant receiving and reacting to external biological and physical factors, i.e., water, light or heat. The deepest layer of the apple peel is the *hypodermis*. It consists of layers of cells located under the *epidermis*, and its main function is to protect the inside of the plant organ from the external environment.

The wax layer and cutin, which are the main components of the cuticle, are susceptible to the penetration of lipophilic compounds, e.g., boscalid, captan, cypermethrin and fludioxonil [40]. On the other hand, polar and water-soluble (hydrophilic) compounds, e.g., cyprodinil, pirimicarb, propiconazole and tebuconazole, may be retained on the surface or can also diffuse through open lenticels located in the fruit peel and via microcracks formed on the apple surface due to the maturation process [41]. Thus, the peel may not be a significant barrier for pesticides, and they can permeate into the pulp. Importantly, pesticide spraying after harvesting or during storage poses a particular risk as the number of microcracks on the surface increases, providing an easy route for compounds to migrate, regardless of their properties.

### 3.2. Method Optimization

In order to optimize the permeation tests, the influence of apple peel thickness and apple pulp composition on the process efficiency was assessed. It was noted that the peel diameter should not be less than 20 mm, because it will not cover the cell opening properly, and it should not be larger than 38 mm due to lateral leakage of the acceptor medium. In addition, the peel should be turned over with the outer layer up and placed at an angle so that no air bubbles accumulate under the membrane. Figure S1 of the Supplementary Materials presents microscopic images of apple peel fragments before testing (a), after 5 h (b) and 24 h (c) of pesticide permeation. Noticeably, the thickness of the apple peel before pesticide application is greater compared to peel thickness after permeation testing. The differences result from the pressure forces of the cell clamping system on the peel and the expansion forces during the dosing of a fresh portion of the acceptor medium. Therefore, the use of peels with a thickness of more than 3 mm is recommended. In order to prevent peel damage, the lowest possible stirring speed was applied.

The next step was to optimize the composition of the acceptor medium to reflect the nature of the apple matrix. Due to the risk of clogging the cell with apple particles, a pulp filtration step was introduced. However, during experimental work, sampling and media replacement ports would still occasionally become clogged after some time, and to prevent this phenomenon, various portions of ultrapure water were added to the apple pulp. A volume of 400 mL per 1 kg of apples ensured the performance of the test and the homogeneity of the composition of the acceptor medium, in accordance with the guidelines for transdermal tests [29]. In addition, the qualitative composition of the filtrate was identical to the pulp of tested apples. Due to the sensitivity of the analytical methodology, the maximum allowable amount of the acceptor medium (1 mL) that could be taken from the Franz cell at one time was selected. 

QuEChERS salt mixtures for general fruits and vegetables with a high water content were used to isolate pesticides and purify extracts. To improve the recovery of pH-dependent pesticides such as captan, citrate buffer salts were used. Therefore, the pH values of the obtained extracts after the first stage were higher than the pH of the matrix (2.9–3.4) and ranged from 5 to 5.5. During extract purification, besides magnesium sulfate, PSA sorbent was also used to remove sugars, organic acids and pigments. In this study, the amounts of the salts used and the volumes of ACN were optimized in terms of the sample volume taken from the cell. The analyses of the third part of each batch of purchased apples were carried out based on the analytical procedure proposed in our previous study [27].

### 3.3. Method Validation

Table 1 summarizes the obtained validation parameters, including linear regression equations for each analyte, coefficients of determination (ranging from 0.9951 (captan) to 0.9996 (boscalid)) and selected ions chosen for monitoring. The first listed ion was used for quantification and the other two for confirmation. In the case of propiconazole and cypermethrin, which exhibit stereoisomerism, all detected peaks corresponding to the isomers were considered. In order to minimize the overestimation of captan concentration levels, the sum of the peak areas corresponding to the parent substance and its thermal degradation product (tetrahydrophthalimide–THPI) were included [27]. To minimize this process and prevent captan degradation in the injection port, a higher-pressure mode was used. This resulted in improved linear fit compared to the parent substance alone.

**Figure 3:**
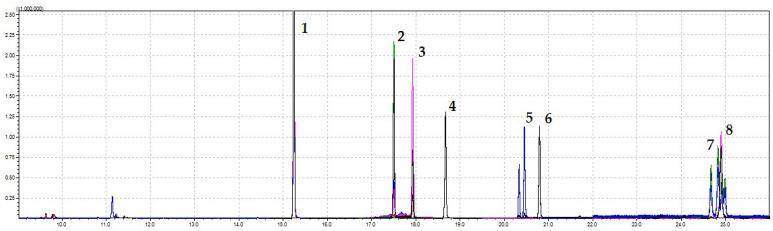
Chromatogram obtained for an apple pulp extract enriched with a mixture of pesticides at 10 mg kg^−1^ concentration level using the QuEChERS–GC-MS/MS methodology; 1—pirimicarb, 2—cyprodinil, 3—captan, 4—fludioxonil, 5—propiconazole, 6—tebuconazole, 7—cypermethrin and 8—boscalid.

The calculated calibration curves showed good linearity from LOQ level up to 10 mg kg^−1^. The coefficient of variability (percentage of relative standard deviation, CV %) was the mean value of different concentrations of the tested pesticides in the linear range and varied from 1.7% (pirimicarb) to 5.6% (captan), which is considered as a good method precision. In addition, the RSD values for the retention time and peak area on two different days were less than 7% and therefore can be considered as satisfactory. The sensitivity of the analytical protocol was considered in terms of LODs, which were in the range from 0.0026 mg kg^−1^ (pirimicarb) to 0.017 mg kg^−1^ (captan). The reproducibility of the method was examined by evaluating the recoveries obtained from the blank samples and samples spiked with standards at 0.1 and 1 mg kg^−1^ concentration levels. As shown in Table 1, the analytes demonstrated good recovery (more than 85%), and the %RSDs were lower than 12%, confirming good accuracy. The results were within acceptable ranges according to guidelines (%RSD less than 20%) [36]. The Matrix effect (ME) was evaluated by comparing the slopes of the calibration curves prepared in apple samples to the one prepared in ACN in the range from −18% (propiconazole) to 19% (boscalid). Figure 3 displays the chromatogram obtained for extract enriched with a mixture of pesticides at a 10 mg kg^−1^ concentration level using the QuEChERS–GC-MS/MS methodology.

### 3.4. Pesticide Permeation through the Peel

**Figure 4:**
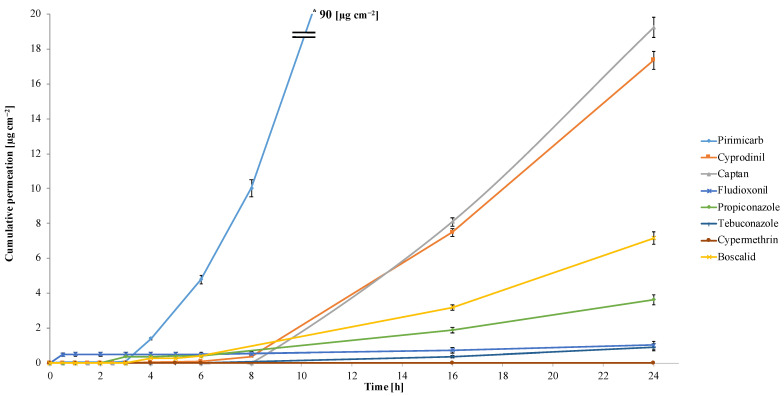
Permeation profiles of the tested pesticides depending on the time elapsed after spraying up to 24 h (presented as the average cumulative amounts expressed in µg per cm^−2^ of apple peel with SD values, n = 3, applied dose of 0.5 mg).

Figure 4 presents the permeation profiles of the tested pesticides depending on the time elapsed after spraying up to 24 h. Based on the obtained data, the highest peel permeability was observed for pirimicarb, captan and cyprodinil. The calculated mean concentration of pirimicarb after 24 h was shown to be the highest and amounted to 23 mg kg^−1^. The result significantly exceeded the linearity range of the developed methodology (10 mg kg^−1^), and the sample was diluted. Therefore, the uncertainty of the obtained result may be relatively higher than for other analytes. The calculated SD from the three measurements was 0.13 mg kg^−1^. The highest diffusion rate of pirimicarb was noted 3 h after spraying, while it was after 6 h for cyprodinil and 8h for captan, respectively. Fludioxonil penetrated the fastest of all studied pesticides. Already 30 min after spraying, its presence in the pulp was observed at the level of 0.12 mg kg^−1^, but the greatest increase in permeation occurred after 8 h, and the final mean concentration in the pulp was 0.26 mg kg^−1^. Tebuconazole exhibited the lowest peel penetration rate and was detected at a 0.096 mg kg^−1^ concentration level 16 h after spraying. Only cypermethrin was not quantitatively detected in the apple pulp after any time interval, even 24 h after spraying. This may result from the physicochemical properties of this compound, i.e., higher molar mass, low water solubility and high log P value. In addition, the study was performed on fresh apples, during their ripening period, and most likely cypermethrin dissolved in the wax layer covering the peel and therefore did not permeate into the pulp. It was observed that the tested compounds penetrated apple peels quantitatively in the following order: cypermethrin < tebuconazole < fludioxonil < propiconazole < boscalid < cyprodinil < captan < pirimicarb. Moreover, no relationship was found between the type of pesticide, its mode of action and penetration results. Both systemic and contact pesticides have shown potential to overcome apple pulp. Systemic pesticides exhibited faster penetration and in higher amounts, with the exception of triazole fungicides. Among the contact pesticides, only captan showed a significant degree of permeation. The proposed methodological solution made it possible to determine not only the ability of selected pesticides to penetrate into the deeper layers of the peel but also to quantify the absorption level in the pulp.

### 3.5. Assessment of Pesticide Absorption

The permeation parameters with toxicological information are shown in Table 2, along with the percentage of total absorption [34]. The statistical evaluation showed that all the values were significant. No calculations were made for cypermethrin, which was not detected in the pulp. The highest cumulative permeations were observed for pirimicarb, captan and cyprodinil with mean values of 90, 19 and 17 µg cm^−2^, respectively. The lowest penetrations were measured for tebuconazole and fludioxonil at the level of 0.91 and 1.1 µg cm^−2^. Pirimicarb has a relatively low molar mass and a relatively high pKa value, illustrating the hydrophilic nature of the compound, which contributes to faster and greater permeability into the apple pulp. An important parameter affecting the results of the study is also the log P coefficient, which is relatively low for pirimicarb. It should be noted that compounds with low log P values are characterized by lower bioaccumulation potentials and are moderately lipophilic. Therefore, compared to other compounds, pirimicarb penetrates fruit peels, covered with hydrophobic waxes, more effectively. Tebuconazole, on the other hand, exhibited the lowest quantitative permeation. This may be influenced by the fact that this pesticide has a relatively high molar mass and high log P. A linear relationship (R^2^ = 0.8319) was observed between the level of pesticide permeation and the reciprocal of the log P value, as shown in Figure S2 of the Supplementary Materials. This points to log P as being one of the most important parameters in determining the process of pesticide absorption, which should be considered when assessing the safety of application to specific crops in order to prevent food contamination.

Total absorption of pirimicarb was 32%, followed by 6.8% and 6.2% for captan and cyprodinil, respectively. Tebuconazole and fludioxonil were characterized by the lowest total absorption, 0.32% and 0.36%, respectively. The exception was propiconazole, with a total absorption of 1.3%, resulting in pulp content exceeding the MRL. Although these compounds permeated in relatively small amounts, their residual levels, which were higher than the MRLs, confirm that the use of traditional removal methods—including peeling (which is generally recognized as a sufficient method of removing residues)—will be ineffective. Where the lowest absorption was observed, the compound was safer to use because less of it remained in the pulp and therefore posed the least risk of exposure. It should be emphasized that in field conditions, pesticide solutions used for spraying may contain different concentration levels of active substances depending on the purpose and type of problem and therefore may be higher than those used in this study. The dose used in this study resulted from the sensitivity and linearity of the analytical methodology in order to enable the determination of the studied compounds at their MRL values. Therefore, the absorption could be even higher.

With regard to pesticides exceeding MRLs, a health risk assessment should be performed to evaluate exposure doses, linking them to the ARfD and ADI values and acting to minimize the related risks [2]. Considering the ARfD doses of pesticides related to short-term exposure, no exceedance was observed for propiconazole. In the case of cyprodinil, health risks may be related to low body weight. For example, for a body weight of 10 kg, the permissible dose is 1 mg, and for 70 kg, it is 7 mg, respectively, assuming that the exposed person eats 1 kg of apples at one time. Therefore, the content in pulp at the level of 4.4 mg will be higher than the allowed doses. The highest short-term risk is posed by pirimicarb at a dose well above the reference dose.

## 4. Conclusions

The results of this study indicate that pesticides have the ability to penetrate apple peels and permeate into the pulp. Therefore, peeling, washing and other traditional removal techniques are insufficient due to the presence of residue in the pulp, which in turn may contribute to the potential risk for consumers. The concentrations of some compounds in apple pulp, 24 h after spraying, were higher than MRLs. The important factors determining pesticide penetration, leading to their presence in the pulp, are exposure time to pesticide spraying, the dose to which the fruit is exposed, the physicochemical properties of the pesticide used and its mode of action, the fruit variety and its ripening process. The peel is one of the pathways of pesticide transport to the pulp, causing contamination that, according to legal regulation, must be monitored.

The Franz diffusion cell system has been successfully used to assess the barrier effect of apple peels. There were no issues with the integrity of the peels during testing, maintaining the set diffusion conditions or measuring precisely defined volumes of the pulp for further quantitative analysis. Therefore, reproducible test results were obtained. Difficulties may arise in the preparation of peels for testing, as some fruits or vegetables (e.g., strawberries, peaches, etc.) have an extremely delicate *epidermis* and may be damaged during the permeation test. Therefore, it may be necessary to apply a thicker layer of peel or another method of cutting. Moreover, the limitation of the proposed method may be the examination of fruit with a smaller diameter, which can be solved using a Franz cell with a correspondingly smaller volume. The modified QuEChERS method in combination with GC-MS/MS has proved to be effective, in terms of specificity, selectivity and accuracy, for the qualitative and quantitative determination of pesticides in apple pulp samples. The developed analytical protocol has allowed the determination of which of the tested pesticides migrate through the peels and which ones only adsorb on their surface. Compounds such as cypermethrin, which do not permeate into the pulp and fulfill their function as intended, do not pose a threat to consumers. They can be effectively removed by washing, peeling, heat treatment or other processes. The obtained data may be helpful in determining the conditions of highest pesticide permeation, which is an important factor in the assessment of acute dietary exposure, as it may lead to a higher intake than previously thought.

## 5. Patents

The solution proposed in this paper is the subject of a patent application in the Polish Patent Office No. [WIPO ST 10/C PL435230].

## Figures and Tables

**Table 1:** Basic validation parameters for individual pesticides obtained using the QuEChERS method and GC-MS/MS.

No.	Analyte	Retention Time [min]	Time Window [min.]	Monitored Ions ^b^ [m/z]	Equation	Coefficient of Determination R^2^	Limit of Detection LOD [mg kg^−1^]	Linearity Range [mg kg^−1^]	Coefficient of Variability CV [%]	Recovery (RSD) [%] of Analyte (n = 5)		Matrix Effect [%]
										0.1 mg kg^−1^	1 mg kg^−1^	0.1 mg kg^−1^
1.	Boscalid	24.89	22.00–26.00	140, 112, 166	y = 5,578,169x – 250,461	0.9996	0.0043	0.013–10	3.9	85 (12)	98 (1.6)	19
2.	Captan	17.92	17.70–18.40	79, 117, 149	y = 3,804,694x + 212,979	0.9951	0.017	0.051–10	5.6	112 (7.8)	103 (2.6)	13
3.	Cypermethrin	24.61–25.05	22.00–26.00	163, 181, 127	y = 1,716,500x + 10,859	0.9992	0.0057	0.017–10	3.3	89 (8.0)	99 (6.9)	11
4.	Cyprodinil	17.50	17.20–17.70	224, 225, 77	y = 9,308,814x − 9998	0.9990	0.0072	0.022–10	3.4	88 (9.1)	97 (4.0)	−12
5.	Fludioxonil	18.67	18.40–19.50	248, 127, 154	y = 3,559,193x – 423,457	0.9993	0.0074	0.022–10	4.8	92 (11)	99 (8.9)	−4.4
6.	Pirimicarb	15.24	14.70–17.20	166, 72, 238	y = 14,653,557x – 96,622	0.9990	0.0026	0.0078–10	1.7	93 (4.7)	97 (5.8)	16
7.	Propiconazole	20.28–20.60	19.50–22.00	173, 175, 259	y = 3,471,709x – 309,348	0.9995	0.0060	0.018–10	3.0	98 (10)	98 (1.2)	−18
8.	Tebuconazole	20.79	19.50–22.00	125, 250, 70	y = 3,441,188x – 515,312	0.9987	0.0044	0.013–10	3.4	93 (6.8)	99 (4.3)	10

Legend: ^b^—quantitation based on first m/z listed; R^2^—coefficient of determination; LOD—limit of detection; CV—coefficient of variability; RSD—the relative standard deviation; n—number of replicates.

**Table 2:** Results of apple peels’ barrier levels for the tested pesticides after 24 h of permeation tests (n = 3, applied dose of 0.5 mg), together with Maximum Residue Limits (MRLs) and toxicological information.

No.	Analyte	Maximum Residue Limits (MRLs) in Apples [mg kg^−1^]	Acceptable Daily Intake (ADI) [mg kg^−1^ Body Weight per Day]	Acute Reference Dose (ARfD) [mg kg^−1^ Body Weight]	Mean Concentration Values ± SD (with *p* Value *) in Apples after 24 h of Permeation [mg kg^−1^] (n = 3)	Cumulative Permeation after 24 h of Permeation [µg cm^−2^]	Absorption Flux [µg cm^−2^ per Hour]	Total Absorption [%]
1.	Boscalid	2.0	0.040	not applicable	1.8 ± 0.011 (*p* = 0.000015)	7.2	0.30	2.5
2.	Captan	10	0.10	0.30	4.8 ± 0.039 (*p* = 0.000011)	19	0.79	6.8
3.	Cypermethrin	1.0	0.0050	0.0050	<LOQ	<LOQ	<LOQ	<LOQ
4.	Cyprodinil	2.0	0.030	not applicable	4.4 ± 0.028 (*p* = 0.000008)	17	0.71	6.2
5.	Fludioxonil	5.0	0.37	not applicable	0.26 ± 0.0074 (*p* = 0.003373)	1.1	0.050	0.36
6.	Pirimicarb	0.5	0.035	0.10	23 ± 0.13 (*p* = 0.000010)	90	3.8	32
7.	Propiconazole	0.010	0.040	0.10	0.92 ± 0.0089 (*p* = 0.000021)	3.6	0.15	1.3
8.	Tebuconazole	0.30	0.030	0.030	0.23 ± 0.0011 (*p* = 0.000031)	0.91	0.040	0.32

Legend: MRL—Maximum Residue Limit; ADI—Acceptable Daily Intake; ARfD—Acute Reference Dose; SD—standard deviation; *—All *p*-values refer to the one-way analysis of variance (ANOVA); n—number of replicates.

## Data Availability

The datasets generated during and/or analyzed during the current study are available from the corresponding author upon reasonable request.

## Supplementary Materials

The following supporting information can be downloaded at https://www.mdpi.com/article/10.3390/foods12173220/s1, Table S1: characteristics and basic properties of the studied pesticides; Figure S1: microscopic images (magnification 40×) of apple peel fragments before testing (a), after 5 h (b) and 24 h (c) of pesticides permeation, obtained with a Nikon Eclipse TS100 F inverted microscope; Figure S2: relationship between the level of permeation after 24 h of spraying and the reciprocal of the log P value of the tested pesticides with SD values, n = 3, applied dose of 0.5 mg.
