# Supplementary material for: Assessment of Apple Peel Barrier Effect to Pesticide Permeation Using Franz Diffusion Cell and QuEChERS Method Coupled with GC-MS/MS
Source: Foods. 2023 Aug 27;12(17):3220. doi: 10.3390/foods12173220 (PMC10486934; doi:10.3390/foods12173220)
Supplement: Supplementary file 1 [file foods-12-03220-s001.zip › foods-2554512-supplementary.pdf]

Supplementary material

Table S1 Characteristics and basic properties of the studied pesticides [34,35]

No	Analyte	Chemical formula	Substance Group	Type of action	Mode of action	Human health issues	Molecular mass [g mol^{-1}]	Solubility in water at 20 °C [mg L^{-1}]	Dissociation constant (pKa) at 25 °C	log P[a] at pH 7, 20 °C
1.	Boscalid	$C_{18}H_{12}Cl_2N_2O$	Carboxamide	Fungicide	Systemic	Toxic to liver and thyroid, possibly carcinogen	343.21	4.6	-	2.96
2.	Captan	$C_9H_8Cl_3NO_2S$	Phthalimide	Fungicide, bactericide	Non-systemic	Inhibition of oestrogen action, skin and eye irritant	300.61	5.2	-	2.5
3.	Cypermethrin	$C_{22}H_{19}Cl_2NO_3$	Pyrethroid	Insecticide	Non-systemic	Highly toxic, estrogenic effect, respiratory tract irritant, eye irritant, possibly toxic to liver and kidney	416.30	0.009	-	5.55
4.	Cyprodinil	$C_{14}H_{15}N_3$	Anilinopyrimidine	Fungicide	Systemic	Skin and eye irritant, respiratory tract irritant	225.29	13	4.44 (weak base)	4.0
5.	Fludioxonil	$C_{12}H_6F_2N_2O_2$	Phenylpyrrole	Fungicide	Non-systemic	Toxic to liver and kidney, skin and eye irritant	248.19	1.8	0 (pKa(1) base; pKa(2) 14.1 acid)	4.12
6.	Pirimicarb	$C_{11}H_{18}N_4O_2$	Carbamate	Insecticide	Systemic	Ache inhibitor, neurotoxic, eye irritant	238.39	3100	4.4 (weak base)	1.7
7.	Propiconazole	$C_{15}H_{17}Cl_2N_3O_2$	Triazole	Fungicide	Systemic	Respiratory tract irritant, liver toxicant, weak oestrogen and aromatase activity inhibition	342.22	150	1.09 (very weak base)	3.72
8.	Tebuconazole	$C_{16}H_{22}ClN_3O$	Triazole	Fungicide, plant growth regulator	Systemic	Eye irritant, reproductive effect, targets liver/blood system	307.82	36	5.0	3.7

Legend:
- no dissociation
[a]Octanol-water partition coefficient at pH 7, 20 °C

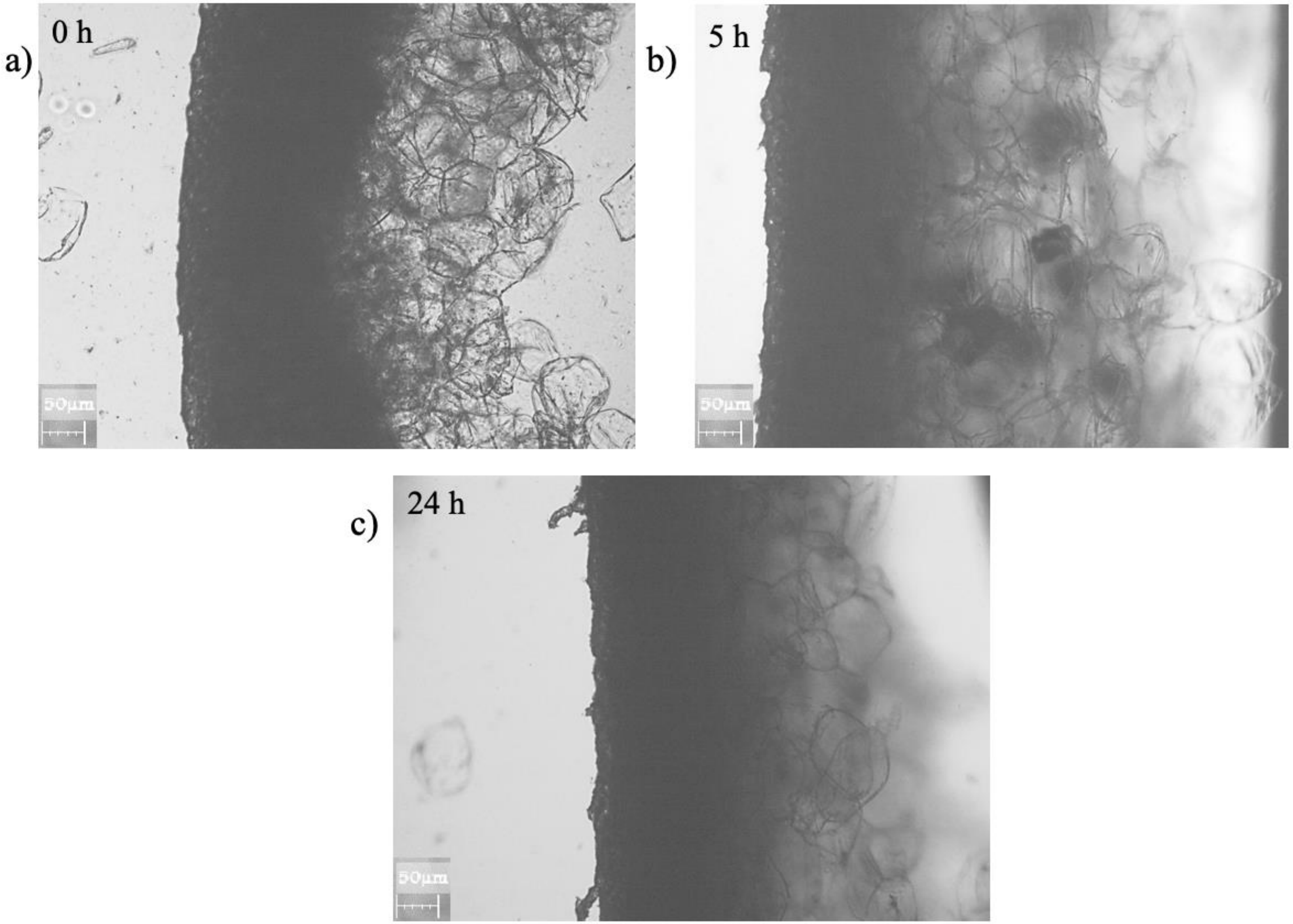

Figure S1 Microscopic images (magnification 40x) of apple peel fragments before testing (a), after 5 hours (b) and 24 hours (c) of pesticides permeation, obtained with a Nikon Eclipse TS100 F inverted microscope

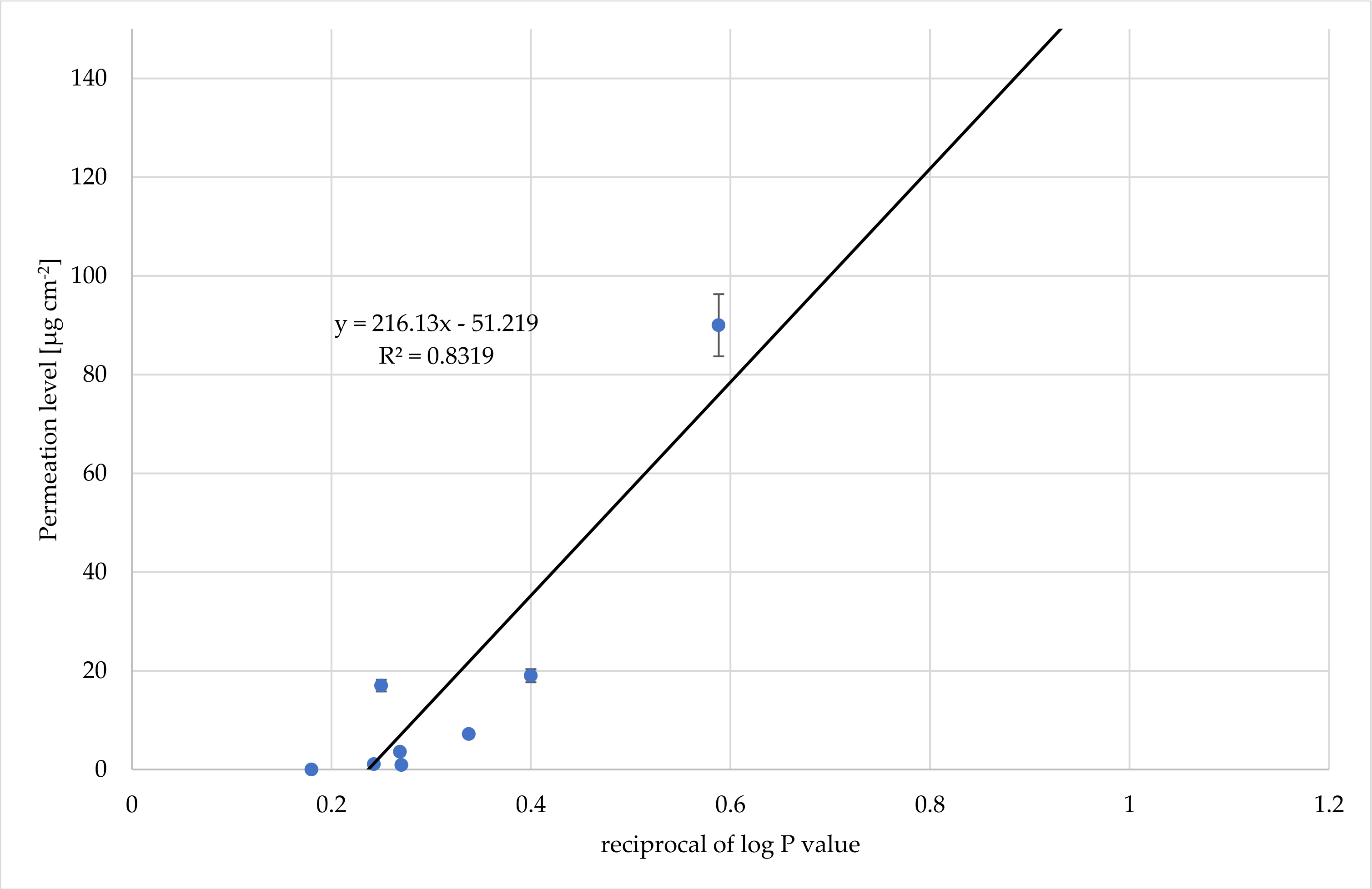

Figure S2 Relationship between the level of permeation after 24 hours of spraying and the reciprocal of the logP value of the tested pesticides with SD values, n=3, applied dose of 0.5 mg
